# The Unexplored Diversity of Pleolipoviruses: The Surprising Case of Two Viruses with Identical Major Structural Modules

**DOI:** 10.3390/genes9030131

**Published:** 2018-02-28

**Authors:** Nina S. Atanasova, Camilla H. Heiniö, Tatiana A. Demina, Dennis H. Bamford, Hanna M. Oksanen

**Affiliations:** 1 Research Programme on Molecular and Integrative Biosciences, Faculty of Biological and Environmental Sciences, University of Helsinki, Viikinkaari 9, FI-00014 Helsinki, Finland; camilla.heinio@helsinki.fi (C.H.H.); tatiana.demina@helsinki.fi (T.A.D.); dennis.bamford@helsinki.fi (D.H.B.); hanna.oksanen@helsinki.fi (H.M.O.); 2 Finnish Meteorological Institute; Erik Palménin aukio 1, FI-00101 Helsinki, Finland

**Keywords:** pleomorphic virus, pleolipovirus, *Pleolipoviridae*, haloarchaeal virus, hypersaline environment, genomic diversity

## Abstract

Extremely halophilic *Archaea* are the only known hosts for pleolipoviruses which are pleomorphic non-lytic viruses resembling cellular membrane vesicles. Recently, pleolipoviruses have been acknowledged by the International Committee on Taxonomy of Viruses (ICTV) as the first virus family that contains related viruses with different DNA genomes. Genomic diversity of pleolipoviruses includes single-stranded and double-stranded DNA molecules and their combinations as linear or circular molecules. To date, only eight viruses belong to the family *Pleolipoviridae*. In order to obtain more information about the diversity of pleolipoviruses, further isolates are needed. Here we describe the characterization of a new halophilic virus isolate, *Haloarcula hispanica* pleomorphic virus 4 (HHPV4). All pleolipoviruses and related proviruses contain a conserved core of approximately five genes designating this virus family, but the sequence similarity among different isolates is low. We demonstrate that over half of HHPV4 genome is identical to the genome of pleomorphic virus HHPV3. The genomic regions encoding known virion components are identical between the two viruses, but HHPV4 includes unique genetic elements, e.g., a putative integrase gene. The co-evolution of these two viruses demonstrates the presence of high recombination frequency in halophilic microbiota and can provide new insights considering links between viruses, membrane vesicles, and plasmids.

## 1. Introduction

Pleolipoviruses are rather newly-described, related pleomorphic viruses that infect extremely halophilic archaeal hosts from the genera *Halorubrum*, *Haloarcula*, or *Halogeometricum* [1]. At the time of writing, the family *Pleolipoviridae* comprises eight members and five related, but unclassified, pleomorphic viruses: *Halorubrum* pleomorphic virus 7 (HRPV-7), HRPV-8, *Haloarcula* pleomorphic virus 2 (HAPV-2), *Natrinema* virus SNJ2, and *Haloarcula hispanica* pleomorphic virus 3 (HHPV3) [1,2,3,4]. The viral family consists of three genera, *Alphapleolipovirus*, *Betapleolipovirus*, and *Gammapleolipovirus*, and is acknowledged by the International Committee on Taxonomy of Viruses (ICTV) as a virus family that contains related viruses with different nucleic acid types [1]. Genomes of the known haloarchaeal pleomorphic viruses are approximately 7–17 kb in size and can be either circular single-stranded (ss) DNA, circular or linear double-stranded (ds) DNA, or circular dsDNA with single-stranded interruptions [1,5]. In addition to the known pleolipoviruses, a number of related proviruses have been identified in the genomes of halophilic *Archaea* [3,4]. These proviruses, as well as all of the known pleolipoviruses, share a conserved core of four to five genes (or open reading frames (ORFs)) with varying degree of sequence similarity. While some of the genes in the conserved core defining the virus family remain unknown, others have been identified encoding, e.g., the viral major structural proteins, which include the spike and internal membrane protein, as well as a putative nucleoside triphosphatase (NTPase) [3,4,6]. 

Aside genomic diversity, one of the unique features of pleolipoviruses is their simplistic virion architecture. Pleolipovirus virions are flexible membranous vesicles which lack a rigid capsid, nucleocapsid, or even nucleoproteins [6,7,8]. Irregularly-distributed spike proteins are embedded in the viral lipid membrane, while internal membrane proteins are located beneath the membrane and have been suggested to interact with the DNA [7]. Pleolipoviruses acquire their lipids unselectively from the host cells, which, together with the non-lytic infection cycle, indicate that virions exit the host cell by budding [6]. Membrane fusion is the most likely entry mechanism of these viruses, but further studies are needed for confirmation. Most pleolipoviruses have a narrow host range and persistent infection cycle, but the rate of host growth retardation has been observed to vary among the different viruses. For example, gammapleolipovirus His2 and betapleolipovirus HHPV3 retard host growth significantly, while betapleolipovirus *Halogeometricum* pleomorphic virus 1 (HGPV-1) infection does not result in detectable host growth retardation [4,6]. The viruses classified into the three different genera of the family *Pleolipoviridae* have been suggested to differ in the mode of replication. Members of the genus *Alphapleolipovirus*, which have either circular ssDNA or dsDNA genomes, have a gene encoding a putative replication initiation protein suggesting that their genomes replicate by rolling circle mechanism [1]. Single-stranded discontinuities observed in the circular dsDNA genomes of betapleolipoviruses may have a role in viral replication [3,5]. The only characterized gammapleolipovirus, His2 with a linear dsDNA genome, has been suggested to use a protein-primed replication mechanism [9]. Although a putative integrase gene has been identified in all the pleolipovirus-like proviruses, only SNJ2 has been shown to integrate into the tRNA^Met^ gene of the host *Natrinema* sp. J7–1 [3]. The integrase of SNJ2 belongs to the tyrosine integrase family and is related to the integrases of the pleolipovirus-like proviruses found in archaeal genomes [3]. Recently, SNJ2 integrase together with over 500 SNJ2-type integrases identified in the genomes of haloarchaea have been shown to constitute a novel family of tyrosine recombinases [10]. Temperate lifestyle is, however, not the only feature of SNJ2 that has brought more information about the diversity of pleolipovirus lifestyles. SNJ2 was observed to have a yet unknown, possibly synergistic, relationship with a temperate icosahedral internal-membrane containing virus SNJ1 [3]. The presence of SNJ1 as a plasmid significantly increases the number of infective SNJ2 progeny from *Natrinema* sp. J7–1 [3]. All the other known pleolipoviruses do not have a putative integrase encoding gene. These viruses have been isolated from hypersaline water or salt crystal samples [2,4,6,9]. The pleolipoviruses with their hosts and the *Archaea* harboring pleolipovirus-like proviruses are found in various hypersaline environments, including solar salterns [2,6,8], saline lakes [6,9], and even deep-sea salt formations [11], indicating worldwide distribution. Like their hosts, pleolipoviruses require high concentrations of NaCl for stability, although the ability to withstand saturated NaCl concentrations varies between these viruses. Members of the genus *Betapleolipovirus* are known to stay infectious even in saturated salt [4]. In addition, HHPV3 stability is also dependent on calcium ions. 

The overall sequence similarity between the different pleolipoviruses is generally low [5], but all of them contain conserved core genes or ORFs and their genomes are collinear [1]. No significant DNA homology is found between pleolipoviruses and bacterial or eukaryotic viruses or even other archaeal viruses [12]. To date the only other known viruses that structurally resemble pleolipoviruses are the mycoplasma phages L2 and L172 [13,14]. The DNA genome sequence of L2 is not related to pleolipoviruses while there is no data about L172 genome sequence [14]. The close resemblance of pleolipovirus virion architecture to membrane vesicles as well as the sequence similarity to various archaeal plasmids has raised interest in the origin and possible co-evolution of viruses, plasmids, and vesicles [12,15]. Membrane vesicles are universally produced by cells belonging to all of the three domains of life [16,17] and have several functions in nature, including transfer of DNA, metabolites, antimicrobials, virulence factors, and signaling molecules [15,18]. Archaeal membrane vesicles are known for instance to contain lipids and S-layer proteins [19]. In addition, among *Sulfolobus* cells, antimicrobials called sulfolobicins have been associated with membranous vesicles produced by these cells [20]. Recently, a so called “infectious” plasmid was isolated from an Antarctic halophilic archaeum *Halorubrum lacusprofundi* R1S1 [21]. The plasmid, pR1SE, can transfer itself inside a membrane vesicle into a plasmid-free strain inducing membrane vesicle production from that strain [21]. Plasmid pR1SE does not contain homologous genes or ORFs with pleolipoviruses or other viruses, except for the putative integrase gene [21].

Here we report the first case of two pleolipoviruses, which have partially 100% identical genomes. A novel, extremely halophilic pleomorphic virus isolate, *H. hispanica* pleomorphic virus 4 (HHPV4), has identical genomic elements encoding major structural modules with those of another pleolipovirus, HHPV3, but its genome is longer and contains a putative integrase gene, which is absent in HHPV3. The striking similarity and coincidental disparity of the two viruses is discussed in terms of new insights in the diversity of vesicle-like viruses in hypersaline environments.

## 2. Materials and Methods 

### 2.1. Strains and Growth Media

The strains used in this study were *Haloferax* sp. s5a–1 [22] and *H. hispanica* American Type Culture Collection, ATCC 33960 [23]. The cells were grown aerobically in modified growth medium (MGM) [24] at 37 °C. Artificial, 30% salt water (SW) that contains 240 g NaCl, 30 g MgCl_2_ × 6H_2_O, 35 g MgSO_4_ × 7H_2_O, 7 g KCl, 5 mL of 1 M CaCl_2_ × 2H_2_O, and 80 mL of 1 M Tris-HCl pH 7.2 (per litre of water) was prepared according to the Halohandbook [25]. One litre of medium contained 18% or 23% SW (top layer); 20% or 23% SW (solid media); and 23% SW (liquid), as well as 5 g of peptone (Oxoid, Thermo Fisher, Waltham, MA, USA) and 1 g of Bacto™ yeast extract (Becton, Dickinson and Company, Franklin Lakes, NJ, USA). Top layer and solid media were prepared by adding 4 g and 14 g of Bacto™ agar (Becton, Dickinson and Company), respectively.

### 2.2. Virus Isolation and Preparation of Virus Stocks

Five-hundred microliters of *H. hispanica* culture at mid-exponential growth phase (optical density at 550 nm, OD_550_ = 1.0) were plated with 3 mL 18% MGM soft agar on a 20% MGM plate, and let to solidify for 1 h at 22 °C. Culture supernatant of *Haloferax* sp. s5a–1 was prepared by removing the cells at the stationary growth phase (OD_550_ = 1.2) by centrifugation (Heraeus Biofuge, Thermo Fisher, 15,700× *g*, 5 min, 22 °C). Ten microliter drops of the culture supernatant (undiluted and 1:100 dilution) were applied on the lawn of *H. hispanica* and incubated at 37 °C for three days. One clear plaque was isolated, purified three consecutive times, and designated as HHPV4. 

Using *H. hispanica* as the host, agar stocks of HHPV4 were prepared from top agar layer of semiconfluent plates. The top layer agar was collected and incubated in MGM liquid broth (3 mL per plate) at 37 °C for 1.5 h with shaking, and debris was removed by centrifugation (Sorvall F14 rotor, Thermo Fisher, 15,191 *g*, 20 min, 4 °C). The agar stocks produced from the plaque isolates derived from the second induction experiment were prepared in the same way.

### 2.3. Sensitivity of Virus Infectivity 

All virus stability tests were performed by diluting virus agar stock 1:1000 in HHPV4 buffer (3.4 M NaCl, 120 mM MgSO_4_, 110 mM MgCl_2_, 70 mM KCl, 4 mM CaCl_2_, 50 mM Tris-HCl, pH 7.2) followed by incubation at 4 °C for 3 h and 24 h, if not specified otherwise. Virus titers were measured by plaque assay. Sensitivity to different ions in the 18% SW used as the basis for HHPV4 buffer was assayed by using 18% SW buffer lacking one or two ionic components at a time. Virus stability at different NaCl concentrations was assayed by using HHPV4 buffer with varying (0–5.0 M) NaCl concentration and incubation time of 7 d at 4 °C. In order to validate the effect of pH, 70 mM 1,4-piperazinediethanesulfonic acid (PIPES), pH 6.1, 50 mM Tris-HCl, pH 7.2, or 70 mM Tris-HCl, pH 9.1 were tested with 1.5 M and 3.4 M NaCl by diluting virus agar stock in the respective buffers and incubating for seven days at 4 °C. Experiments were performed three times. 

The effect of CaCl_2_ on HHPV4 infectivity was tested by using HHPV4 buffer containing 0–8 mM CaCl_2_. In addition, a chelating agent ethylene glycol-bis(β-aminoethyl ether)-N,N,N′,N′-tetraacetic acid (10 mM EGTA) was used to remove residual CaCl_2_ in HHPV4 buffer devoid of CaCl_2_. Experiments were performed twice. Sensitivity to sucrose and CsCl was assayed by using 60% (*w*/*v*) sucrose solution or CsCl solution (ρ = 1.3 g/mL) prepared in HHPV4 buffer. Sensitivity to Triton X-100 (Merck, Darmstadt, Germany) and Nonidet P40 (LKB Bromma, Stockholm, Sweden) was determined by incubating 0.1 and 0.01% (*v*/*v*) final concentrations of the detergents with the agar stock for 15 min at 22 °C. Chloroform sensitivity was assayed by adding chloroform (Merck) to HHPV4 agar stock in the ratio 1:4 and incubating for 15 min at 22 °C.

### 2.4. Virus Adsorption and Life Cycle

For virus adsorption assay, *H. hispanica* cells (OD_550_ = 1.0) were collected by centrifugation (Sorvall F14 rotor, Thermo Fisher, 7450× *g*, 22 °C, 15 min) and concentrated ten-fold by suspending into fresh MGM at 37 °C. The culture was infected with HHPV4 using a multiplicity of infection (MOI) of 0.14 and grown aerobically at 37 °C. Samples were collected until 4 h post infection (p.i.), diluted in ice-cold MGM (1:100), centrifuged (Heraeus Biofuge, Thermo Fisher, 15,700× *g*, 1 min, 22 °C), and the titer of the supernatant was determined by plaque assay. The adsorption rate constant (*k*) was calculated according to the formula

$$k=\frac{2.3}{B\times t}\times log\frac{p0}{p}$$

where *p*0 and *p* represent free infectious virus concentrations at the time of infection and after time period *t*, respectively. *B* equals the concentration of the viable cells [26]. The experiment was repeated three times.

In order to measure virus release in liquid culture, a MOI of 15 was used to infect *H. hispanica* cells at OD_550_ = 1.0 (1.5 × 10^9^ colony-forming unit (cfu)/mL). The number of free progeny viruses was measured at 24 h post infection (p.i.) from the culture supernatant. The cells were washed twice at 2.5 h p.i., by collecting the cells by centrifugation (Sorvall SA600 rotor, Thermo Fisher, 9300× *g*, 15 min, 22 °C) and resuspending them into the original volume of fresh MGM. The number of infective centres and viable cells was measured from 0 to 2.5 h p.i. at 30 min intervals by collecting the cells by centrifugation (Heraeus Biofuge, Thermo Fisher, 15,700× *g*, 5 min, 22 °C) and resuspending in fresh MGM. The washing of the cells was repeated twice. The number of free progeny viruses in the culture supernatant was determined at 4–30 h p.i. by plaque assay simultaneously monitoring the turbidity (OD_550_) of the infected and uninfected (control) cultures. The experiment was repeated three times.

### 2.5. Virus Purification

HHPV4 particles were precipitated from agar stocks using 10% (*w*/*v*) polyethylene glycol (PEG) 6000 (Ubichem). The mixture was incubated at 4 °C for 1 h with shaking. The precipitate was collected by centrifugation (Sorvall F14 rotor, Thermo Fisher, 9700× *g*, 40 min, 4 °C) and resuspended into HHPV4 buffer at 4 °C (~1:40 of the original culture volume). Viruses were either purified once (1× purified virus) or twice (highly purified; 2× purified virus). To obtain 1× purified virus, the PEG-precipitated particles were subjected to ultracentrifugation in linear 10–40% (*w*/*v*) sucrose gradients (HHPV4 buffer; Sorvall TH641 rotor, Thermo Fisher, 106,900× *g*, ~20 h, 15 °C or Sorvall AH629 rotor, Thermo Fisher, 83,600× *g*, 17 h, 15 °C). The 1× purified virus particles in the light-scattering zone were diluted 1:2 into HHPV4 buffer, concentrated by differential centrifugation (Sorvall T647.5 rotor, Thermo Fisher, 93,300× *g*, 2–3 h, 15 °C), and resuspended into HHPV4 buffer. In order to obtain 2× purified virus, the 1× purified virus light scattering zone was diluted 1:2 in HHPV4 buffer and purified by ultracentrifugation in linear 20–60% (*w*/*v*) sucrose gradients (HHPV4 buffer; Sorvall TH641 rotor, Thermo Fisher, 106,900× *g*, ~20 h, 20 °C or Sorvall AH629 rotor, Thermo Fisher, 83,600× *g*, 17 h, 20 °C). The 2× purified virus particles in the light scattering zone were diluted 1:2 in HHPV4 buffer and concentrated by differential centrifugation (Beckman Ti50 rotor, (Beckman Coulter Inc., Brea, CA, USA) 93,800× *g*, 2.5 h, 20 °C or T647.5 rotor, 99,800× *g*, 3 h, 15 °C) and resuspended into HHPV4 buffer. HHPV3 particles were produced and purified according to the 1× purification protocol by [4]. Protein concentrations were measured by the Coomassie Blue method [27] with bovine serum albumin as a standard. Sodium dodecyl sulfate polyacrylamide gel electrophoresis (SDS-PAGE) gels contained 16% (*w*/*v*) acrylamide in the separation gel [28]. Proteins were visualized by Coomassie Brilliant Blue R 250 (Serva, Heidelberg, Germany) staining. To detect lipids in the SDS-PAGE gels, the gels were stained with Sudan Black B (Sigma Aldrich, St. Louis, MO, USA) according to the manufacturer’s instructions.

### 2.6. Analysis of Extracted Lipids

*H. hispanica* cells were collected from liquid culture (OD_550_ = 1.0) and concentrated 100-fold. Concentrated cells were resuspended into HHPV4 buffer and used for lipid extraction. Highly-purified 2× HHPV4 virus particles were used for lipid extraction. Lipid extraction was performed according to the protocol by Folch et al. [29] as modified by Kates in 1972 [30]. The extracted lipids were analysed using pre-activated thin layer chromatography (TLC) silica plates and ammonium molybdate staining for visualization [31]. The plates were stained with a solution containing 10% (*v*/*v*) H_2_SO_4_ and 5% (*w*/*v*) ammonium molybdate. The excess liquid on the plate was dried followed by a 15 min incubation at 140 °C. 

### 2.7. Negative Stain Electron Microscopy 

Five microliters of the 2× HHPV4 virus particles were incubated on copper pioloform-coated grids for 1 min at 22 °C. The particles were negatively stained with 1% (*w*/*v*) ammonium molybdate for 1 min. Transmission electron microscopy (TEM) was performed by a JEOL 1400 transmission electron microscope (Electron Microscopy Unit, HiLIFE-Institute of Biotechnology, University of Helsinki, Helsinki, Finland) operating at 80 kV acceleration voltage. Virion diameter was determined as an average diameter of 12 particles measured in one TEM micrograph.

### 2.8. Genome Isolation and Sequencing

Nucleic acid was extracted from the 1× HHPV4 virus particles that were diluted with deionized water and treated with proteinase K (0.5 mg/mL, Thermo Fisher) and 2% (*w*/*v*) SDS for 45 min at 37 °C. Phenol-ether extraction of the nucleic acid was followed by precipitation by NaCl and ethanol. The purified nucleic acid was treated with RQ1 DNase (Promega; Madison, WI, USA), RNase A (Fermentas, Thermo Fisher), Exonuclease III (Fermentas), Bal31 nuclease (New England Biolabs, Ipswich, MA, USA), as well as with restriction enzymes: AseI, SalI, HincII, MseI, SspI, SmaI, MscI, NruI (New England Biolabs), NotI (Fermentas), and HindIII (Roche, Basel, Switzerland) according to the manufacturer’s instructions. 

Untreated virus DNA, DNA treated with *Sulfolobus* polymerase IV and T4 ligase, and DNA treated with Klenow fragment and T4 ligase were pooled together and sequenced using Illumina MiSeq (Illumina, San Diego, CA, USA). The reads were trimmed using cutadapt (1.9) with minimum length of 200 bp and minimum quality of 30. Adapter sequences were removed using cutadapt (1.9) [32]. Reads were assembled using SPAdes 3.1.1 [33]. HHPV4 genome sequence has been deposited in the GenBank database under the accession number KY264020.

### 2.9. Genome Annotation

Geneious version 6.1.8 [34] was used for the HHPV4 genome sequence analyses. Ori-Finder 2 [35] was used to obtain the HHPV4 genome sequence disparity curves and to identify putative origin recognition box (ORB) sequences. ORFs were predicted using Glimmer [36], GeneMarkS [37], and FGENESV (http://www.softberry.com/), with the preference towards predictions that are in collinearity with the previously annotated related virus genomes. GC content of the predicted ORFs was calculated with Science Buddies Genomics %G~C Content Calculator (http://www.sciencebuddies.org/science-fair-projects/project_ideas/Genom_GC_Calculator.shtml). The molecular weight (MW) and isoelectric point (pI) of the (putative) proteins were calculated using the Compute pI/Mw tool from ExPASy [38]. Transmembrane helices in (putative) proteins were predicted using the TMHMM Server v. 2.0 [39]. Coiled-coils were predicted with the COILS program from ExPASy [40]. Signal peptides were predicted using the Phobius webserver [41]. Blastx (search dated 3/10/2016) was used for aligning HHPV4 sequences against protein sequences deposited in the NCBI non-redundant protein database [42]. BLASTP (Delta-blast) (search dated 22 November 2016) [43] and Interproscan (search dated 7 November 2016) [44] were used to detect putative conserved domains and other protein signatures. EMBOSS Needle from EMBL-EBI [45] was used for global alignment of nucleotide/protein sequences. The Mauve alignment algorithm was used for whole genome alignments [46].

### 2.10. Plaque Purification and PCR

A new set of six plaques were isolated as described above and plaque-purified three consecutive times. Their similarity to HHPV4 was assayed by PCR using HHPV4-specific primers (forward: CTCCGGACCAGTGACGAAAA; reverse: ACGAACGTGTCCTGATTGCT) matching to a region in gene 11 which encodes the spike protein. For PCR, the plaques purified three consecutive times were incubated in deionized water overnight at 4 °C, treated with 10% (*w*/*v*) SDS, boiled for 5 min, diluted with deionized water (1:10), and used as templates. A single HHPV4 plaque, as well as purified HHPV4 DNA, were used as positive controls. 

In order to find out whether HHPV4 sequence could be located from *H. hispanica* or *Haloferax* sp. s5a-1, separate colonies of these strains were isolated, diluted with 50 µL of deionized water, boiled for 5 min, centrifuged (15,700× *g*, 5 min, 5 °C, Eppendorf centrifuge 5415D) and the supernatant was diluted 1:2, 1:5, or 1:10 in deionized water, and used as a template. PCR with HHPV4-specific primers (see above) and primers D30 and D56 [47] for 16S rRNA gene (as a positive control) was performed.

### 2.11. Phylogenomic Analysis

Phylogenomic analysis was performed using the VICTOR online resource (http://ggdc.dsmz.de/victor.php). All pairwise comparisons of the nucleotide and amino acid sequences were conducted using the Genome-BLAST Distance Phylogeny (GBDP) method [48] under settings recommended for prokaryotic viruses [49]. The resulting intergenomic distances (including 100 replicates each) were used to infer a balanced minimum evolution tree with branch support via FASTME including SPR (Surface Plasmon Resonance) post processing [50] for each of the formulas D0, D4, and D6, respectively. When nucleotide sequences were analysed (all genomes completely sequenced), the distance formula D0 was preferred, while in amino acid sequences analysis, the formula D6 was preferred, as recommended in [49].

The trees were rooted at the midpoint [51] and visualized with FigTree [52]. Taxon boundaries at the species, genus, and family level were estimated with the OPTSIL program [53], the recommended clustering thresholds [49] and an F value (fraction of links required for cluster fusion) of 0.5 [54].

## 3. Results

### 3.1. HHPV4 Was Isolated From Spot Assay Plates of *H. hispanica*

A hazy plaque was isolated from the lawn of *H. hispanica* which had been inoculated with the culture supernatant of *Haloferax* sp. s5a–1. The virus was designated as *H. hispanica* pleomorphic virus 4 (HHPV4). Titers of HHPV4 agar stocks reached approximately 10^11^ plaque-forming unit (pfu)/mL and were stable at 4 °C for several months (data not shown).

In order to confirm that the culture supernatant of *Haloferax* sp. s5a–1 induces plaque production, the spot assay was repeated, and plaques with similar appearance to those produced by HHPV4, were observed on the lawn of *H. hispanica*. Six plaques were plaque-purified, and in all cases, a specific PCR product with HHPV4-specific primers was obtained confirming that the induced plaques were produced by HHPV4. Titers of the agar stocks produced from the purified plaque isolates (the second induction) were similar to that of HHPV4. The virus did not infect *Haloferax* sp. s5a–1. 

### 3.2. Virus Infectivity Depends on Salt Concentrations 

**Figure 1:**
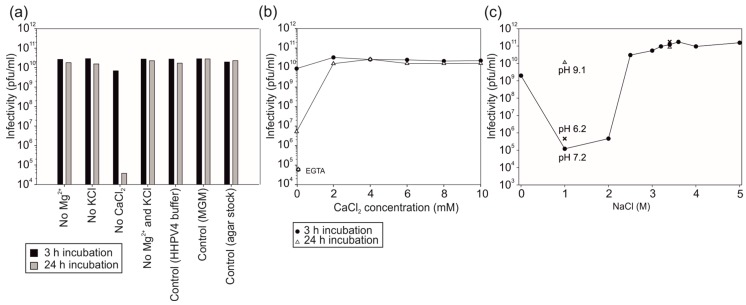
*Haloarcula hispanica* pleomorphic virus 4 (HHPV4) infectivity in different ionic conditions. (**a**) Infectivity in HHPV4 buffer (3.4 M NaCl, 120 mM MgSO_4_, 110 mM MgCl_2_, 70 mM KCl, 4 mM CaCl_2_, 50 mM Tris-HCl, pH 7.2) from which one or two components at a time have been removed. Infectivity was assayed after 3 h (black bars) and 24 h (grey bars) incubation at 4 °C. Modified growth medium (MGM) and agar stock were used as controls; (**b**) The effect of CaCl_2_ concentration in HHPV4 buffer on virus infectivity after 3 h (circles) and 24 h (triangles) incubation at 4 °C. Chelating agent EGTA was used to remove residual CaCl_2_; (**c**) The effect of NaCl concentration in HHPV4 buffer (pH 7.2) on virus infectivity after 7 d incubation at 4 °C (circles). Virus infectivity in HHPV4 buffer at pH 6.2 (crosses) and pH 9.1 (triangles) in the presence of 1 M and 3.4 M NaCl were assayed in the same incubation conditions. Pfu, plaque-forming unit.

Removal of different ionic compounds from the virus buffer one or two at a time indicated that infectivity of HHPV4 is sensitive to the absence of CaCl_2_ (Figure 1a) and at least 2 mM CaCl_2_ is required for stability (Figure 1b). A high concentration of NaCl was needed to maintain virus infectivity, and HHPV4 was shown to be sensitive to any concentration lower than 3 M (Figure 1c). In addition, the infectivity was the lowest at 1–2 M NaCl (a reduction of 5–6 orders of magnitude), but dependent on the pH used (Figure 1c). HHPV4 infectivity remained high at 1–2 M NaCl concentration at pH 9.1, as opposed to pH values 6.1 or 7.2, which inactivated the viruses (Figure 1c). The other tested ions did not affect virus infectivity at detectable levels (Figure 1a). According to these results, the composition of the HHPV4 buffer was designed as the following: 3.4 M NaCl, 120 mM MgSO_4_, 110 mM MgCl_2_, 70 mM KCl, 4 mM CaCl_2_, 50 mM Tris-HCl, pH 7.2. 

HHPV4 infectivity dropped at least two orders of magnitude when the particles were treated with chloroform. Treatment with the nonionic detergents, Triton X-100 and Nonidet P40, resulted in a total loss of infectivity suggesting the presence of lipids in the HHPV4 virion. The virus titers dropped one order of magnitude after incubation in CsCl solution (ρ = 1.3 g/mL in HHPV4 buffer) while dense sucrose (60% (*w*/*v*)) did not affect infectivity. Consequently, sucrose was chosen as the gradient material for virus purification.

### 3.3. HHPV4 Infection Leads to High Progeny Production

**Figure 2:**
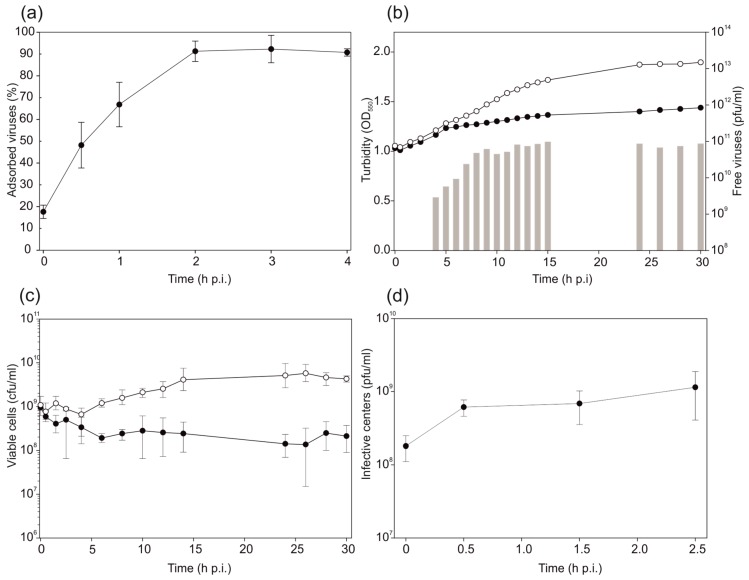
Virus adsorption and life cycle. (**a**) Adsorption of HHPV4 to *Haloarcula hispanica* cells at 37 °C. Error bars represent one standard deviation (*n* = 3); (**b**–**d**) Life cycle of HHPV4 in *H. hispanica*; (**b**) Turbidity of the infected (black circles) and uninfected (white circles) *H. hispanica* cultures and the number of free infectious viruses (grey bars); (**c**) Viable cells of the infected (black circles) and uninfected (white circles) cultures; (**d**) Infective centres of the infected *H. hispanica* culture for the first 2.5 h post infection (p.i.). Error bars represent one standard deviation in (**c**) and (d) (*n* = 3). Cfu, colony-forming unit.

Approximately 90% of HHPV4 particles adsorbed to *H. hispanica* cells at 2 h p.i. (Figure 2a). The adsorption rate constant was 1.7 × 10^−12^ mL/min for the first 30 min p.i. In the optimized virus life cycle experiment, MOI of 15 was used to infect *H. hispanica* culture when optical density (OD_550_) was ~1.0. The highest increase in the production of free progeny viruses was observed starting from 4 h p.i. (Figure 2b). At the same time, the growth rate of the infected culture started to decline based on the turbidity measurements. At 8 h p.i., a stable production of ~10^11^ pfu/mL was reached (Figure 2b). The number of viable cells started to diverge between the control and the infected cultures starting at 6 h p.i. (Figure 2c), after which the viable cells in the infected culture reached the concentration of ~10^8^ cfu/mL for the rest of the measurements, while, in the control, the numbers increased and remained above 10^9^ cfu/mL. The number of infective centres reached ~10^9^ pfu/mL at 2.5 h p.i., indicating that a high number of host cells were infected by HHPV4 (Figure 2d).

### 3.4. Two-Step Virus Purification Yielded a High Number of Pure Infectious Particles 

**Figure 3:**
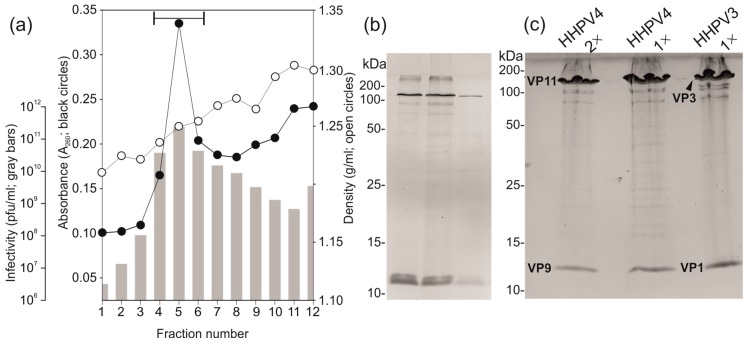
HHPV4 purification and structural proteins. (**a**) Infectivity (grey bars), absorbance (black circles), and density (g/mL; open circles) of the 20–60% (*w*/*v*) sucrose gradient fractions (2× purified HHPV4 particles). Fraction numbers correspond to 4 mL fractions (AH629 tube). No. 1 is the top of the gradient. The position of the light scattering zone with the highest infectivity is indicated by a horizontal segment of a line; (**b**) The protein pattern of the virus peak analysed by SDS-PAGE and Coomassie Blue staining (the position of the segment of a line in a). (**c**) Comparison of the protein profiles of the 1× and 2× purified HHPV4 to that of the 1× purified HHPV3 (Coomassie Blue stained SDS-PAGE gel). HHPV4 proteins (virion proteins, VP) VP9 and VP11, as well as the corresponding HHPV3 proteins VP1 and VP3 [4], are indicated.

Viruses were purified by a two-step (1× and 2× purification) sucrose gradient purification protocol, as described in Materials and Methods. Virus purification resulted in highly-pure infectious particles with a yield of up to 15% (Table S1). After 2× purification, the highest concentration of infectious viruses was observed at a density of ~1.27 g/mL (sucrose) in line with the absorbance peak (Figure 3a). The calculated specific infectivity of HHPV4 was ~4.9 × 10^13^ pfu/mg of protein indicating high purity and stable integrity of the pure virions. The protein profile of the HHPV2 virions was simple (Figure 3b), which was found to be very similar to that of the HHPV3 virus (Figure 3c) [4]. The two major structural proteins of HHPV4, designated as VP9 (VP for virion protein; ~120 kDa) and VP11 (~13 kDa) (see below), correspond to the HHPV3 proteins VP1 and VP3, respectively (Figure 3c). 

### 3.5. HHPV4 is a Pleomorphic, Membrane-Containing Virus

**Figure 4:**
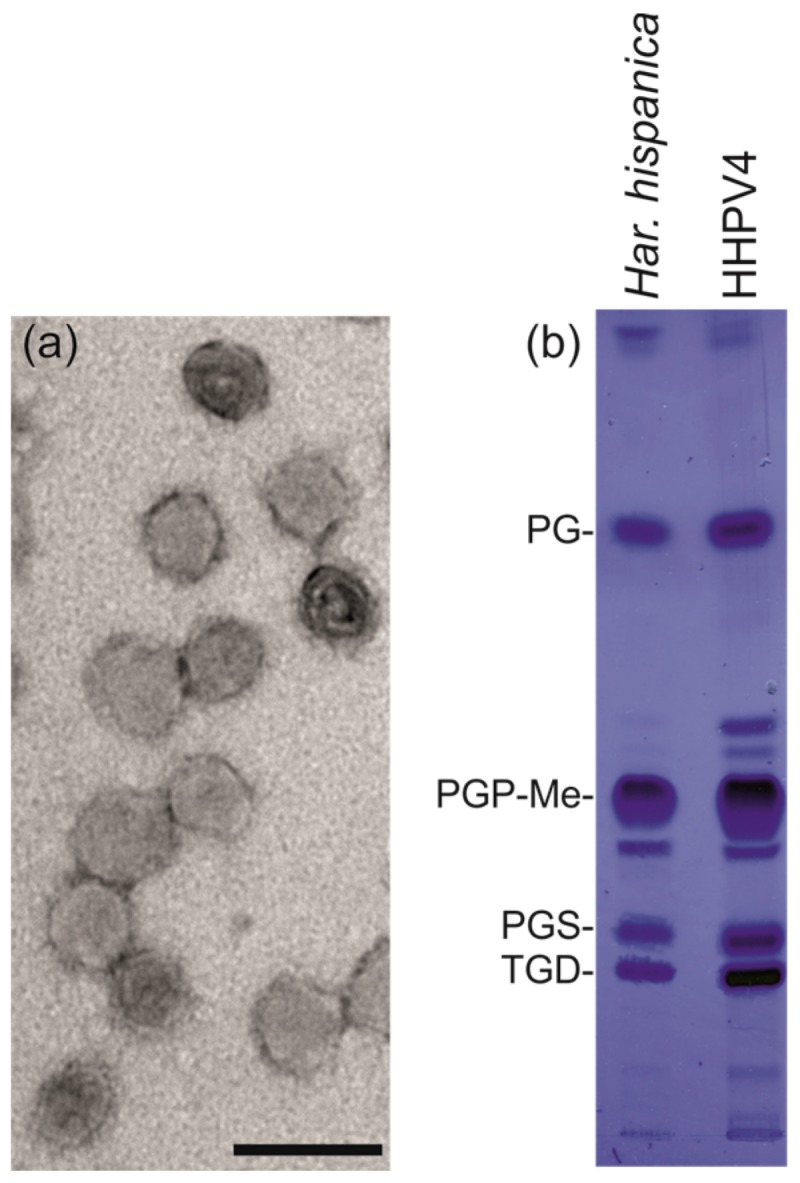
HHPV4 virion and its lipids. (**a**) Transmission electron microscopy (TEM) micrograph of the 2× purified HHPV4 particles negatively stained with 1% ammonium molybdate. Scale bar equals 100 nm; (**b**) Thin-layer chromatogram of lipids extracted from *H. hispanica* cells and 2× purified HHPV4 particles. The major lipid species of *H. hispanica* [55] are indicated on the left: PG, phosphatidylglycerol; PGP-Me, phosphatidylglycerophosphate methyl ester; PGS, phosphatidylglycerosulfate; TGD, triglycosyl glycerodiether.

The TEM micrographs of the 2× purified HHPV4 particles indicate spherical or pleomorphic morphology (Figure 4a). The mean virion diameter was ~60 nm. Sudan Black staining of the 2× purified particles separated on an SDS-PAGE gel suggested that HHPV4 particles contain lipids (Figure S1). Lipids extracted from the 2× purified viruses and *H. hispanica* cells revealed similar lipid profiles, indicating that the virus acquires its lipids unselectively from the host (Figure 4b). Based on the known lipid species of *H. hispanica* [55], the HHPV4 membrane is composed of at least phosphatidylglycerol (PG), phosphatidylglycerophosphate methyl ester (PGP-Me), phosphatidylglycerosulfate (PGS), and triglycosyl glycerodiether (TGD) (Figure 4b).

### 3.6. The HHPV4 Genome Is a Circular dsDNA Molecule

The HHPV4 genome was resistant to RNase A, but was degraded by RQ1 DNase, Exonuclease III, and cleaved by a number of restriction enzymes (*Ase*I, *Sal*I, *Hinc*II, *Mse*I, *Ssp*I, and *Sma*I) (Figure S2a), showing that it is a dsDNA molecule. When HHPV4 DNA was treated with Exonuclease III (Figure S2a) or Bal31 nuclease (Figure S2b), a pattern of multiple fragments was observed, suggesting the presence of single-stranded interruptions. The GCCCA motif associated with single-stranded gaps/nicks in the genomes of HRPV3 [5] and SNJ2 [3] can also be found in the HHPV4 genome (35 motifs) (Figure S3a). Sequencing of the DNA molecule revealed that it is 15,010 bp in length, and the assembly suggested a circular conformation. When HHPV4 DNA was treated with the restriction enzymes MscI and HindIII (both are single-cutters for HHPV4), only one fragment was visible in the gel, while after incubation with NruI (having three cutting sites), three fragments of the expected sizes (~1.8, ~2.4, and ~10.8 kb) were observed (Figure S2c), confirming that HHPV4 DNA is circular.

The RY base disparity curve (the distribution of purine versus pyrimidine nucleotides) of the HHPV4 sequence obtained using Ori-Finder 2 programme, [35] had two transition sites (a minimum at between 5000 and 6000 bp and a maximum at ~11,000 bp), which may correspond to the replication origin and terminus (Figure S3). Conserved ORB sequences were found close to the curve minimum (Table S2).

### 3.7. The HHPV4 Genome Sequence Is Largely Identical to That of HHPV3 and Contains a Conserved Pleolipoviral Block

**Figure 5:**
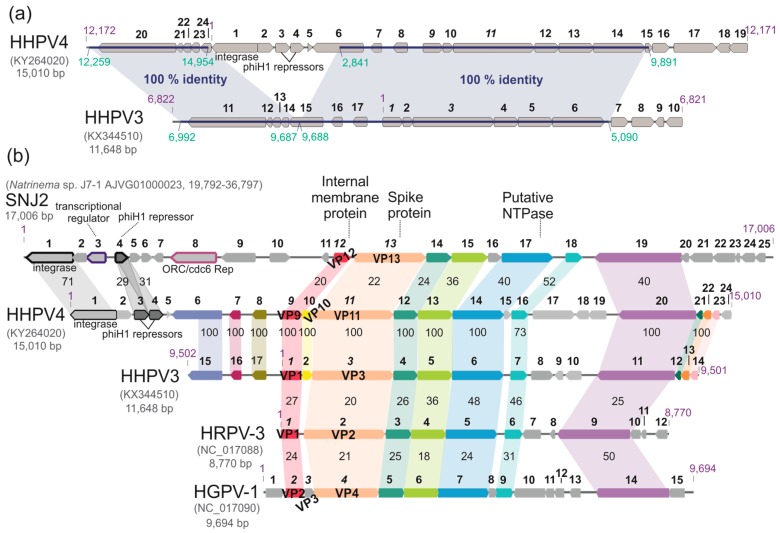
Comparison of genomes of betapleolipoviruses. All genomes are circular double stranded (ds) DNA molecules, which are shown as linearized molecules. Open reading frames (ORFs) or genes are represented as grey arrows and are numbered (gene numbers are italicized). GenBank accession numbers are indicated in brackets, and the total length of genome sequences is marked under virus name. Nucleotide coordinates are shown in purple. (**a**) Comparison of HHPV4 and HHPV3 genomes. The regions of 100% nucleotide identity are marked with blue, and nucleotide coordinates of the identical regions are shown in dark green; (**b**) All known betapleolipovirus genomes are compared. Virion proteins (VPs) are marked. Similar ORFs or genes are in the same colours. Amino acid identities (%) between (putative) proteins are shown in between the genomes. HRPV-3, *Halorubrum pleomorphic* virus 3; HGPV-1, *Halogeometricum* pleomorphic virus 1.

**Figure 6:**
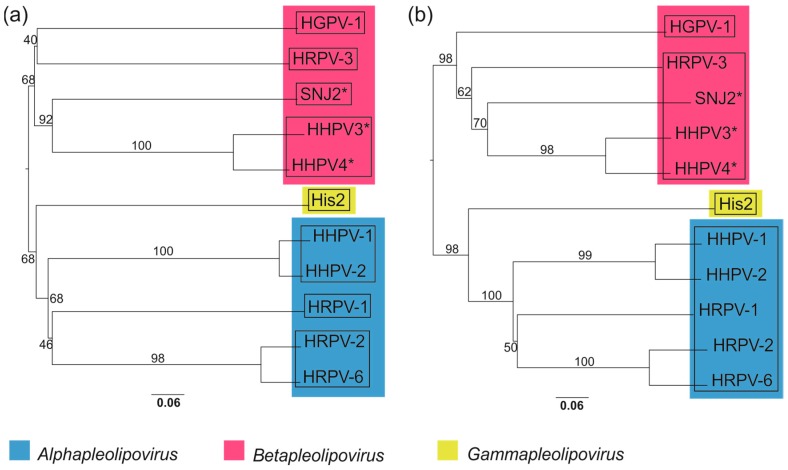
Phylogenomic Genome BLAST Distance Phylogeny (GBDP) trees of the whole genomic sequences of the members and putative members of the family *Pleolipoviridae* at the (**a**) nucleotide and (**b**) amino acid levels. Viruses classified or proposed to be classified (marked by asterisks) into three genera of the family *Pleolipoviridae* are marked with different colours (see the colour code). Genera yielded by OPTSIL clustering are shown as squares. The branch lengths are scaled in terms of the GBDP distance formula D0 in (**a**) and the GBDP distance formula D6 in (**b**) [49]. The trees and OPTSIL clusters were obtained using the VICTOR web service [49].

Surprisingly, a substantial part of the HHPV4 genome sequence is identical to that of pleolipovirus HHPV3, which also infects *H. hispanica* (Figure 5a). Two regions in the HHPV4 genome (nucleotides (nt) 12,259–14,954 and nt 2841–9891) are identical to the regions in one continuous stretch in the HHPV3 genome (nt 6992–9687 and nt 9688–11,648, 1–5090) (Figure 6a). These identical regions cover, in total, ~65% and ~84% of the HHPV4 and HHPV3 genome sequences, respectively. 

Twenty-four ORFs were predicted in the HHPV4 genome, coding for putative proteins of 38-613 amino acid residues (4–65 kDa) with pI of 4–6, in most cases (Table S3). ORFs or genes (and their corresponding proteins) were numbered from 1–24 starting from the ORF encoding for a putative integrase (see below). A conserved block of ORFs or genes characteristic to pleolipoviruses was identified in the HHPV4 genome (ORFs or gene nos. 9–14, 16, and 20; Figure 5b). All ORFs or genes belonging to this conserved block are identical in HHPV4 and HHPV3, except HHPV4 putative protein 16 and HHPV3 putative protein 7, sharing ~73% amino acid identity. HHPV4 genes *9* and *11* are 100% identical to HHPV3 genes *1* and *3*, respectively, and are thus confirmed to be genes coding for the major structural proteins VP9 and VP11 (Figure 3c). Based on the similarity to HHPV3 and other pleolipovirus sequences, HHPV4 VP9 is the membrane-associated small structural protein, while VP11 is the spike protein. VP9 contains four predicted transmembrane helices, and for VP11, a putative signal peptide (27 first amino acid residues) was identified (Table S3). HHPV4 putative protein VP10, which is predicted to contain a transmembrane helix, shows ~19% identity and ~36% similarity at the amino acid level with pleolipovirus His2 protein VP27, which is a membrane-associated protein located at the internal side of the membrane bilayer [6]. This suggests that HHPV4 has two membrane protein species, VP9 and VP10, which are encoded by two neighbouring genes. The HHPV4 putative protein VP10 is identical to HHPV3 putative protein 2 (Figure 5a) suggesting that HHPV3 also has two small membrane protein types. It has been previously shown that pleolipovirus HGPV-1 also has two membrane protein types [6]. The HHPV4 putative protein 14, contains a predicted AAA-ATPase domain (Table S4), suggesting that this protein is most probably an NTPase, for which conserved counterparts have been found in all pleolipoviruses (Figure 5b). 

### 3.8. HHPV4 Genome Differs from That of HHPV3 by the Presence of an Integrase-Encoding Gene

Based on similarity searches and detected conserved domains (Table S4), HHPV4 putative protein 1 is predicted to be an integrase (which is ~71% identical to SNJ2 integrase at the amino acid level), and putative proteins 3 and 4 are PhiH1-like repressors (Figure 5). These proteins, together with putative proteins 2, 5, and (partially) 6 are encoded by ORFs that are found in the HHPV4 genetic module between the genomic regions identical to the HHPV3 sequence (Figure 5a). HHPV4 putative proteins 3 and 4 share ~28% amino acid identity, and both are ~30% identical to a PhiH1-like repressor protein identified in a temperate pleolipovirus SNJ2 (Figure 5b). HHPV4 ORF6 locates on the border between HHPV4-specific and HHPV3-identical regions, and it is partly identical to HHPV3 ORF15 (Figure 5b). HHPV4 putative protein 6 is predicted to be a restriction endonuclease, as it has a restriction endonuclease domain (Dam-replacing family, like in HHPV3) and an HNH endonuclease domain (Table S4). 

### 3.9. HHPV4-Like Proviruses Are Detected in Haloarchaeal Genomes

HHPV4-related sequences are found in many haloarchaea as previously described [3,4,5,8,9,56,57,58]. We have identified one new putative provirus in the genome of *Haloarcula* sp. K1 (contig 6 NZ_LRHL01000043.1, reversed) (Figure S4). As in the other cases, this putative provirus is also flanked by the integrase gene and the tRNA gene. 

Interestingly, the predicted integrase and PhiH1-like repressor of HHPV4 are ~95% and ~98% identical to the counterparts found in a pleolipovirus-related provirus in *Haloarcula marismortui* ATCC 43049 region 1 [3,4]. A number of other putative proteins are also highly similar between HHPV4 (and HHPV3) and this putative *H. marismortui* provirus [4]. However, the whole HHPV4 genome sequence cannot be found in any of the published sequences, including those of *H. hispanica* ATCC 33960 (GenBank acc. nos. NC_015948.1, NC_015943.1, NC_015944.1) or *H. hispanica* N601 (NC_023013.1, NC_023010.2, NC_023012.1, NC_023011.1). In addition, no PCR product was obtained using colonies of *H. hispanica* or *Haloferax* sp. s5a-1 as templates with the HHPV4-specific primers, while the archaeal 16S ribosomal RNA gene primers used as positive controls resulted in PCR products (data not shown).

### 3.10. Whole Genome-Based Analysis Reinforces the Current Classification of Pleolipoviruses

The Genome BLAST Distance Phylogeny (GBDP)-based phylogenomic trees of the complete genome sequences of the pleolipoviruses yielded average support of ~76% and ~84% at the nucleotide and amino acid levels, respectively (Figure 6a,b). Both trees have two major branches comprising viruses that are currently classified as alphapleolipoviruses and gammapleolipovirus (lower branch, Figure 6a,b) and betapleolipoviruses (upper branch, Figure 6a,b) by the ICTV. As expected, HHPV4 clusters with betapleolipoviruses and most closely with HHPV3. 

Based on nucleotide sequences, the clustering by OPTSIL yielded eleven species (i.e., each virus isolate forming own species), eight genera (Figure 6a, squares), and two families: one for His2 and the other one containing the rest of the proposed virus species. With amino acid sequences, the clustering suggested also eleven species, but four genera (Figure 6b, squares) within one family. Thus, the results based on amino acid sequences correlate rather well with the current taxonomic classification of the pleolipoviruses in the three genera within the family Pleolipoviridae. However, according to the OPTSIL clustering, HGPV-1 should be classified into its own genus, while currently it belongs to the *Betapleolipovirus* genus together with HRPV-3 and presumably HHPV3 [4], SNJ2 [3], and HHPV4.

## 4. Discussion

The recently-described archaeal pleolipoviruses, together with a few bacterial plasmaviruses, are the only known viruses to date that architecturally resemble cellular membrane vesicles that are known to be commonly produced by the members of all three domains of cellular life. The enveloped, quasi-spherical virion architecture, as well as various functions, such as continuous, non-lytic replication style, ability of some of the viruses to integrate in, and excise from, the host chromosome, as well as the non-selective acquisition of host lipids upon virus assembly, all resemble features associated with membranous vesicles or mobile genetic elements [12]. 

Here we present the microbiological and genomic characterization of a new pleolipovirus, HHPV4. The virus adsorption rate constant is similar to that described for HHPV3 (2.4 × 10^–11^ mL/min), although the release of new progeny starts approximately 2 h earlier for HHPV4 (Figure 2a) compared to HHPV3 [4]. The HHPV4 life cycle is non-lytic, as described for other pleolipoviruses [4,6]. Like HHPV3, HRPV-3, HRPV-6, and His2, HHPV4 retards host growth significantly. The number of progeny HHPV4 viruses reaches approximately 10^11^ pfu/mL, which is a considerably high yield produced in liquid culture. Similar values have been obtained for HHPV3, but, interestingly, the numbers of progeny HHPV3 viruses exceed the level of 10^10^ pfu/mL at around 9 h p.i. [4], while for HHPV4 this value is achieved already at around 6 h p.i (Figure 2a). The time points when viable cell counts in the infected culture are significantly declined are in line with the increase in progeny release for both, HHPV3 and HHPV4 (Figure 2b,c) [4]. Surprisingly, we found that over 60% of the HHPV4 genome sequence is identical to that of the previously-described HHPV3, and vice versa, more than 80% of HHPV3 genome is covered by the identical part of the HHPV4 sequence ([4]; Figure 5). Notably, the HHPV3-HHPV4 pair represents the first known example of such closely-related pleolipoviruses. Both viruses infect the extremely halophilic archaeon *H. hispanica*. The genome sequence of HHPV4 cannot be found from *H. hispanica* chromosomes and, except for HHPV3, the virus is not closely related to any previously-described virus or provirus. All the ORFs or genes characteristic to betapleolipoviruses are 100% conserved in HHPV4 and HHPV3, except HHPV4 ORF16 and HHPV3 ORF 7 (73% amino acid identity). Thus far, the function of HHPV4 ORF16 (or HHPV3 ORF7) is not known, and it would be intriguing to see why they are more divergent than the other betapleolipovirus-specific ORFs or genes in these two viruses. 

Although HHPV4 and HHPV3 genome sequences have substantially long identical and collinear parts, these virus genomes clearly differ by the presence of a putative integrase gene and a stretch of ORFs between the ORF encoding a putative NTPase (ORF14 in HHPV4) and the long reverse ORF specific for all betapleolipoviruses (ORF20 in HHPV4). The latter region is less conserved in all betapleolipoviruses (Figure 5), while an integrase-containing module is present only in SNJ2 and HHPV4. The integrase-encoding genetic module found in HHPV4 is likely a result of a recent horizontal gene transfer event, since no point mutations are detected in the long identical genomic regions flanking this module. The presence of an integrase-encoding ORF suggests that HHPV4 might be able to integrate into the host genome. Since no HHPV4-specific sequence was found in its host *H. hispanica* sequence [59], and the virus produces visible plaques on the host lawn, some other *Archaea* may harbor HHPV4 sequences as an integrated virus. Alternatively, HHPV4 integrase may be non-functional. The ORF2 and ORF3 of SNJ2 were recently shown to be critical for SNJ2 integration into the host chromosome [10]. Homologs of these ORFs were found in only five of the pleolipovirus-like proviruses identified in haloarchaeal genomes [10]. No such homologs were detected in the genome of HHPV4. The presence of an integrase gene in SNJ2, HHPV4, as well as numerous related proviruses [3,10], suggests that lysogenic life cycle may also be common for pleolipoviruses. Furthermore, SNJ2-type integrases have also been suggested to have an important role in DNA recombination [10].

Both HHPV3 and HHPV4 have similar requirements for NaCl and CaCl_2_, and an ability to tolerate saturated NaCl concentration, indicating that their virions are both very stable in extremely halophilic environments [4]. Notably, unlike HHPV3 [4], HHPV4 was found to be sensitive to CsCl and had to be purified in dense sucrose gradients instead. The pleomorphic HHPV4 virion is approximately 60 nm in diameter, being larger than the HHPV3 virions which have a 50 nm diameter [4], correlating to the genome sizes of 15,010 bp and 11,648 bp of HHPV4 and HHPV3, respectively [4]. Previous research has shown that pleolipoviruses can package either circular ssDNA or dsDNA, or linear dsDNA [5]. Here, we demonstrate that pleomorphic virus assembly with smaller and larger genomes using the identical protein and lipid components, in both cases, results in functional virions of different sizes. This elegantly illustrates the dynamic assembly process of a pleomorphic virion, which most probably occurs as a co-assembly of the membrane and the genome. The differences in virion and genome sizes as well as sequence lengths, allow speculation about whether HHPV3 and HHPV4 are different virus species that could have evolved from one parental virus by recombination. Alternatively, these two viruses could be considered as variants of the same virus due to the long identical genomic regions. We favor the hypothesis that HHPV4 lost the integrase containing gene block on the course of time resulting in HHPV3, as betapleolipoviruses can function without this element. The ORFs 16 and 17 as well as part of ORF 15 in HHPV3 could, thus, be remnants of these recombination events (Figure 5B). Since HHPV3 and HHPV4 have identical spike proteins, they presumably bind to the same receptor on the host cell surface, indicating possibilities for further rearrangements. The extensive host range tests performed for HHPV3 demonstrate that, from the 47 tested haloarchaeal strains, the virus is specific to *H. hispanica* [4]. The identical spike proteins indicate that HHPV4 also has a narrow host range. 

Genomic and phylogenetic analyses indicate that HHPV4 belongs to the genus *Betapleolipovirus* of the *Pleolipoviridae* family, which also includes the previously-characterized viruses HRPV-3 and HGPV-1, as well as the related, but unclassified, viruses SNJ2 and HHPV3 [1,3,4]. Members of the *Pleolipoviridae* family are specifically known for their genomic diversity. While alphapleolipoviruses are known for having either ssDNA or dsDNA genomes, betapleolipoviruses are distinguished by the peculiar single-stranded interruptions along their circular dsDNA genomes, which are also likely present in the genome of HHPV4 [1,5]. Similarly, site-specific nicks have been detected in the DNA genomes of some bacteriophages, e.g., T5 [60] and T7 [61], but their functions are also unknown. More studies on this unusual type of genome organization are needed to answer the question about what role single-stranded interruptions may have in virus life cycle. 

The whole-genome sequence comparisons, such as phylogenomic analysis using the VICTOR online resource [49], represent a promising approach for sequence-based virus classification. Noticeably, the phylogenetic OPTSIL clustering generally supports current ICTV classification (approved or proposed) within this viral group, except that HGPV-1 is suggested to be placed into yet another genus. His2 remains the sole representative of the genus *Gammapleolipovirus* and the only pleolipovirus with a linear DNA genome and a gene for protein-primed DNA replication [1,9]. More pleolipovirus isolates would help in understanding the genomic diversity of this virus group and yield more accurate classifications.

Pleolipoviruses are widespread in nature and have been isolated from hypersaline water, salt crystals, or archaeal cells derived from Southern Europe, Israel, China, Thailand, or Australia [2,3,4,6,22]. The *Archaea* that are either hosts of the previously described pleolipoviruses or harbor pleolipovirus-like proviruses in their genomes, are even more geographically distributed, as hypersaline environments are found on all the continents of the world [1,3,22]. The most recent observation regarding related proviruses was surprisingly obtained from the genome of *Halanaeroarchaeum sulfurireducens* M27-SA2, which was isolated from a depth of three kilometers in the anoxic, hypersaline Lake Medee located at the Eastern Mediterranean [11]. Here, one more pleolipovirus-like provirus was identified in the genome of strain *Haloarcula* sp. K1. Although sequence similarities between the isolated pleolipoviruses are typically low, the pleolipovirus-specific ORFs in HHPV3 or HHPV4 are more than 90% identical to their counterparts detected in the putative proviruses [4]. This emphasizes that the genetic diversity of pleolipoviruses is understudied, that these viruses are abundant in various distant hypersaline environments, and that there are both distant and close relatives among them. 

The case of HHPV3 and HHPV4 illustrates frequent recombination among the microbiota of hypersaline environments. Recent observations suggest that the genetic relatedness between certain non-viral mobile genetic elements, proviruses, and archaeal viruses could indicate that these viruses possibly obtained important genes related to virion replication or assembly from non-viral origin [10,12]. The recently discovered “infectious” plasmid pR1SE, on the other hand, suggests that membrane vesicles can also have an “infective nature” [21]. The remarkable ability to package variable genomic DNA molecules is characteristic to the archaeal pleomorphic viruses and raises questions about the difference of a pleomorphic virion and an “infective” plasmid packaged inside a membrane vesicle. It is expected that further studies on pleolipoviruses will provide more information about the relationships and possible links between membrane vesicles, genetic elements, and viruses. 

## Supplementary Materials

The following are available online at http://www.mdpi.com/2073-4425/9/3/131/s1. Figure S1. SDS-PAGE analysis of the 2× purified HHPV4 particles; Figure S2. HHPV4 genome treated with nucleases or restriction enzymes, analyzed in agarose gels stained with ethidium bromide; Figure S3. Identified genes and predicted ORFs in the HHPV4 genome; Figure S4. Putative HHPV4-like provirus in the genome of *Haloarcula* sp. K1, Table S1. HHPV4 virus purification statistics; Table S2. Origin recognition box (ORB) sequences found with the Ori-Finder 2 program for patterns specific for *Halobacteriaceae*; Table S3. HHPV4 predicted ORFs and identified genes; Table S4. Putative conserved domains detected in the HHPV4 putative proteins.

## References

[1] Bamford D.H., Pietilä M.K., Roine E., Atanasova N.S., Dienstbier A., Oksanen H.M. (2017). ICTV Report Consortium. ICTV Virus Taxonomy Profile: Pleolipoviridae. J. Gen. Virol..

[2] Atanasova N.S., Demina T.A., Buivydas A., Bamford D.H., Oksanen H.M. (2015). Archaeal viruses multiply: Temporal screening in a solar saltern. Viruses.

[3] Liu Y., Wang J., Liu Y., Wang Y., Zhang Z., Oksanen H.M., Bamford D.H., Chen X. (2015). Identification and characterization of SNJ2, the first temperate pleolipovirus integrating into the genome of the SNJ1-lysogenic archaeal strain. Mol. Microbiol..

[4] Demina T.A., Atanasova N.S., Pietilä M.K., Oksanen H.M., Bamford D.H. (2016). Vesicle-like virion of *Haloarcula hispanica* pleomorphic virus 3 preserves high infectivity in saturated salt. Virology.

[5] Senčilo A., Paulin L., Kellner S., Helm M., Roine E. (2012). Related haloarchaeal pleomorphic viruses contain different genome types. Nucleic Acids Res..

[6] Pietilä M.K., Atanasova N.S., Manole V., Liljeroos L., Butcher S.J., Oksanen H.M., Bamford D.H. (2012). Virion architecture unifies globally distributed pleolipoviruses infecting halophilic *Archaea*. J. Virol..

[7] Pietilä M.K., Laurinavičius S., Sund J., Roine E., Bamford D.H. (2010). The single-stranded DNA genome of novel archaeal virus *Halorubrum* pleomorphic virus 1 is enclosed in the envelope decorated with glycoprotein spikes. J. Virol..

[8] Pietilä M.K., Roine E., Paulin L., Kalkkinen N., Bamford D.H. (2009). An ssDNA virus infecting *Archaea*: a new lineage of viruses with a membrane envelope. Mol. Microbiol..

[9] Bath C., Cukalac T., Porter K., Dyall-Smith M.L. (2006). His1 and His2 are distantly related, spindle-shaped haloviruses belonging to the novel virus group, *Salterprovirus*. Virology.

[10] Wang J., Liu Y., Liu Y., Du K., Xu S., Wang Y., Krupovic M., Chen X. (2018). A novel family of tyrosine integrases encoded by the temperate pleolipovirus SNJ2. Nucleic Acids Res..

[11] Messina E., Sorokin D.Y., Kublanov I.V., Toshchakov S., Lopatina A., Arcadi E., Smedile F., La Spada G., La Cono V., Yakimov M.M. (2016). Complete genome sequence of ‘*Halanaeroarchaeum sulfurireducens*’ M27-SA2, a sulfur-reducing and acetate-oxidizing haloarchaeon from the deep-sea hypersaline anoxic lake Medee. Stand. Genomic Sci..

[12] Krupovic M., Cvirkaite-Krupovic V., Iranzo J., Prangishvili D., Koonin E.V. (2017). Viruses of *Archaea*: Structural, functional, environmental and evolutionary genomics. Virus Res..

[13] Dybvig K., Nowak J.A., Sladek T.L., Maniloff J. (1985). Identification of an enveloped phage, mycoplasma virus L172, that contains a 14-kilobase single-stranded DNA genome. J. Virol..

[14] Dybvig K., Maniloff J. (1983). Integration and lysogeny by an enveloped mycoplasma virus. J. Gen. Virol..

[15] Nolte-’t Hoen E., Cremer T., Gallo R.C., Margolis L.B. (2016). Extracellular vesicles and viruses: Are they close relatives?. Proc. Natl. Acad. Sci. USA.

[16] Soler N., Krupovic M., Marguet E., Forterre P. (2015). Membrane vesicles in natural environments: A major challenge in viral ecology. ISME J..

[17] Deatherage B.L., Cookson B.T. (2012). Membrane vesicle release in bacteria, eukaryotes, and *Archaea*: A conserved yet underappreciated aspect of microbial life. Infect. Immun..

[18] Gaudin M., Gauliard E., Schouten S., Houel-Renault L., Lenormand P., Marguet E., Forterre P. (2013). Hyperthermophilic *Archaea* produce membrane vesicles that can transfer DNA. Environ. Microbiol. Rep..

[19] Ellen A.F., Albers S.V., Huibers W., Pitcher A., Hobel C.F., Schwarz H., Folea M., Schouten S., Boekema E.J., Poolman B. (2009). Proteomic analysis of secreted membrane vesicles of archaeal *Sulfolobus* species reveals the presence of endosome sorting complex components. Extremophiles.

[20] Prangishvili D., Holz I., Stieger E., Nickell S., Kristjansson J.K., Zillig W. (2000). Sulfolobicins, specific proteinaceous toxins produced by strains of the extremely thermophilic archaeal genus *Sulfolobus*. J. Bacteriol..

[21] Erdmann S., Tschitschko B., Zhong L., Raftery M.J., Cavicchioli R. (2017). A plasmid from an Antarctic haloarchaeon uses specialized membrane vesicles to disseminate and infect plasmid-free cells. Nat. Microbiol..

[22] Atanasova N.S., Roine E., Oren A., Bamford D.H., Oksanen H.M. (2012). Global network of specific virus-host interactions in hypersaline environments. Environ. Microbiol..

[23] Juez G., Rodriguez-Valera F., Ventosa A., Kushner D.J. (1986). *Haloarcula hispanica* spec. nov. and *Haloferax gibbonsii* spec. nov., two new species of extemely halophilic archaebacteria. Syst. Appl. Microbiol..

[24] Nuttall S.D., Dyall-Smith M.L. (1993). HF1 and HF2: Novel bacteriophages of halophilic *Archaea*. Virology.

[25] Dyall-Smith M. (2009). The Halohandbook: Protocols for Haloarchaeal Genetics.

[26] Adams M.H. (1959). Bacteriophages.

[27] Bradford M.M. (1976). A rapid and sensitive method for the quantitation of microgram quantities of protein utilizing the principle of protein-dye binding. Anal. Biochem..

[28] Olkkonen V.M., Bamford D.H. (1989). Quantitation of the adsorption and penetration stages of bacteriophage φ6 infection. Virology.

[29] Folch J., Lees M., Sloane Stanley G.H. (1957). A simple method for the isolation and purification of total lipides from animal tissues. J. Biol. Chem..

[30] Kates M. (1972). Techniques of Lipidology: Isolation, Analysis and Identification of Lipids.

[31] Arnold H.P., Zillig W., Ziese U., Holz I., Crosby M., Utterback T., Weidmann J.F., Kristjanson J.K., Klenk H.P., Nelson K.E. (2000). A novel lipothrixvirus, SIFV, of the extremely thermophilic crenarchaeon *Sulfolobus*. Virology.

[32] Martin M. (2011). Cutadapt removes adapter sequences from high-throughput sequencing reads. EMBnet J..

[33] Bankevich A., Nurk S., Antipov D., Gurevich A.A., Dvorkin M., Kulikov A.S., Lesin V.M., Nikolenko S.I., Pham S., Prjibelski A.D. (2012). SPAdes: A new genome assembly algorithm and its applications to single-cell sequencing. J. Comput. Biol..

[34] Kearse M., Moir R., Wilson A., Stones-Havas S., Cheung M., Sturrock S., Buxton S., Cooper A., Markowitz S., Duran C. (2012). Geneious Basic: An integrated and extendable desktop software platform for the organization and analysis of sequence data. Bioinformatics.

[35] Luo H., Zhang C.T., Gao F. (2014). Ori-Finder 2, an integrated tool to predict replication origins in the archaeal genomes. Front. Microbiol..

[36] Delcher A.L., Bratke K.A., Powers E.C., Salzberg S.L. (2007). Identifying bacterial genes and endosymbiont DNA with Glimmer. Bioinformatics.

[37] Besemer J., Lomsadze A., Borodovsky M. (2001). GeneMarkS: A self-training method for prediction of gene starts in microbial genomes. Implications for finding sequence motifs in regulatory regions. Nucleic Acids Res..

[38] Gasteiger E., Gattiker A., Hoogland C., Ivanyi I., Appel R.D., Bairoch A. (2003). ExPASy: The proteomics server for in-depth protein knowledge and analysis. Nucleic Acids Res..

[39] Krogh A., Larsson B., von Heijne G., Sonnhammer E.L. (2001). Predicting transmembrane protein topology with a hidden Markov model: application to complete genomes. J. Mol. Biol..

[40] Lupas A., Van Dyke M., Stock J. (1991). Predicting coiled coils from protein sequences. Science.

[41] Käll L., Krogh A., Sonnhammer E.L. (2007). Advantages of combined transmembrane topology and signal peptide prediction--the Phobius web server. Nucleic Acids Res..

[42] Altschul S.F., Gish W., Miller W., Myers E.W., Lipman D.J. (1990). Basic local alignment search tool. J. Mol. Biol..

[43] Boratyn G.M., Schaffer A.A., Agarwala R., Altschul S.F., Lipman D.J., Madden T.L. (2012). Domain enhanced lookup time accelerated BLAST. Biol. Direct.

[44] Jones P., Binns D., Chang H.Y., Fraser M., Li W., McAnulla C., McWilliam H., Maslen J., Mitchell A., Nuka G. (2014). InterProScan 5: Genome-scale protein function classification. Bioinformatics (Oxford, England).

[45] Rice P., Longden I., Bleasby A. (2000). EMBOSS: The European molecular biology open software suite. Trends Genet..

[46] Darling A.C., Mau B., Blattner F.R., Perna N.T. (2004). Mauve: Multiple alignment of conserved genomic sequence with rearrangements. Genome Res..

[47] Arahal D.R., Dewhirst F.E., Paster B.J., Volcani B.E., Ventosa A. (1996). Phylogenetic analyses of some extremely halophilic *Archaea* isolated from Dead Sea water, determined on the basis of their 16S rRNA sequences. Appl. Environ. Microbiol..

[48] Meier-Kolthoff J.P., Auch A.F., Klenk H.-P., Göker M. (2013). Genome sequence-based species delimitation with confidence intervals and improved distance functions. BMC Bioinformatics.

[49] Meier-Kolthoff J.P., Goker M. (2017). VICTOR: Genome-based phylogeny and classification of prokaryotic viruses. Bioinformatics.

[50] Lefort V., Desper R., Gascuel O. (2015). FastME 2.0: A Comprehensive, Accurate, and Fast Distance-Based Phylogeny Inference Program. Mol. Biol. Evol..

[51] Farris J.S. (1972). Estimating Phylogenetic Trees from Distance Matrices. Am. Naturalist.

[52] Rambaut A. (2006). FigTree 1.4.0—A Graphical Viewer of Phylogenetic Trees and a Program for Producing Publication-Ready Figures.

[53] Göker M., Garcia-Blazquez G., Voglmayr H., Telleria M.T., Martin M.P. (2009). Molecular taxonomy of phytopathogenic fungi: A case study in Peronospora. PLoS ONE.

[54] Meier-Kolthoff J.P., Hahnke R.L., Petersen J., Scheuner C., Michael V., Fiebig A., Rohde C., Rohde M., Fartmann B., Goodwin L.A. (2014). Complete genome sequence of DSM 30083 T, the type strain (U5/41 T) of *Escherichia coli*, and a proposal for delineating subspecies in microbial taxonomy. Stand. Genomic Sci..

[55] Bamford D.H., Ravantti J.J., Rönnholm G., Laurinavičius S., Kukkaro P., Dyall-Smith M., Somerharju P., Kalkkinen N., Bamford J.K. (2005). Constituents of SH1, a novel lipid-containing virus infecting the halophilic euryarchaeon *Haloarcula hispanica*. J. Virol..

[56] Chen S., Wang C., Xu J.P., Yang Z.L. (2014). Molecular characterization of pHRDV1, a new virus-like mobile genetic element closely related to pleomorphic viruses in haloarchaea. Extremophiles.

[57] Dyall-Smith M.L., Pfeiffer F., Klee K., Palm P., Gross K., Schuster S.C., Rampp M., Oesterhelt D. (2011). *Haloquadratum walsbyi*: Limited diversity in a global pond. PLoS ONE.

[58] Roine E., Kukkaro P., Paulin L., Laurinavičius S., Domanska A., Somerharju P., Bamford D.H. (2010). New, closely related haloarchaeal viral elements with different nucleic acid types. J. Virol..

[59] Liu H., Wu Z., Li M., Zhang F., Zheng H., Han J., Liu J., Zhou J., Wang S., Xiang H. (2011). Complete genome sequence of *Haloarcula hispanica*, a Model Haloarchaeon for studying genetics, metabolism, and virus-host interaction. J. Bacteriol..

[60] Abelson J., Thomas C. (1966). The anatomy of the T5 bacteriophage DNA molecule. J. Mol. Biol..

[61] Khan S.A., Hayes S.J., Wright E.T., Watson R.H., Serwer P. (1995). Specific single-stranded breaks in mature bacteriophage T7 DNA. Virology.

