# Supplementary material for: The Unexplored Diversity of Pleolipoviruses: The Surprising Case of Two Viruses with Identical Major Structural Modules
Source: Genes (Basel). 2018 Feb 28;9(3):131. doi: 10.3390/genes9030131 (PMC5867852; doi:10.3390/genes9030131)
Supplement: Supplementary file 1 [file genes-09-00131-s001.pdf]

Supplementary Materials

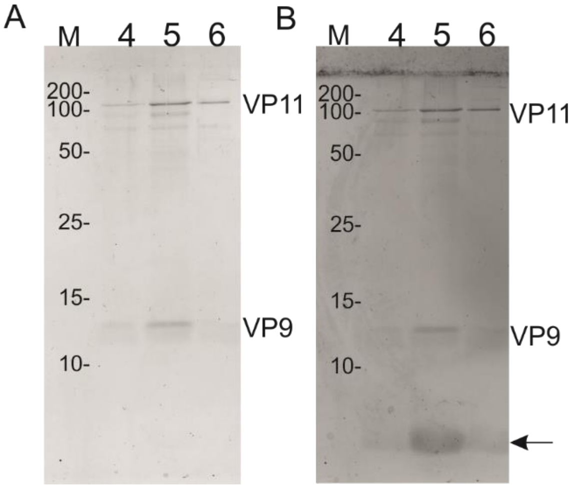

Figure S1. SDS-PAGE analysis of the 2× purified HHPV4 particles with (**A**) Coomassie Brilliant Blue or (**B**) Sudan Black B staining. The peak fractions (nos. 4–6) of 20-60% sucrose gradient are shown (see Figure 3A). The major structural proteins, VP9 and VP11 are marked. Lipids are indicated with an arrow. Positions of the molecular mass standards are shown in kDa.

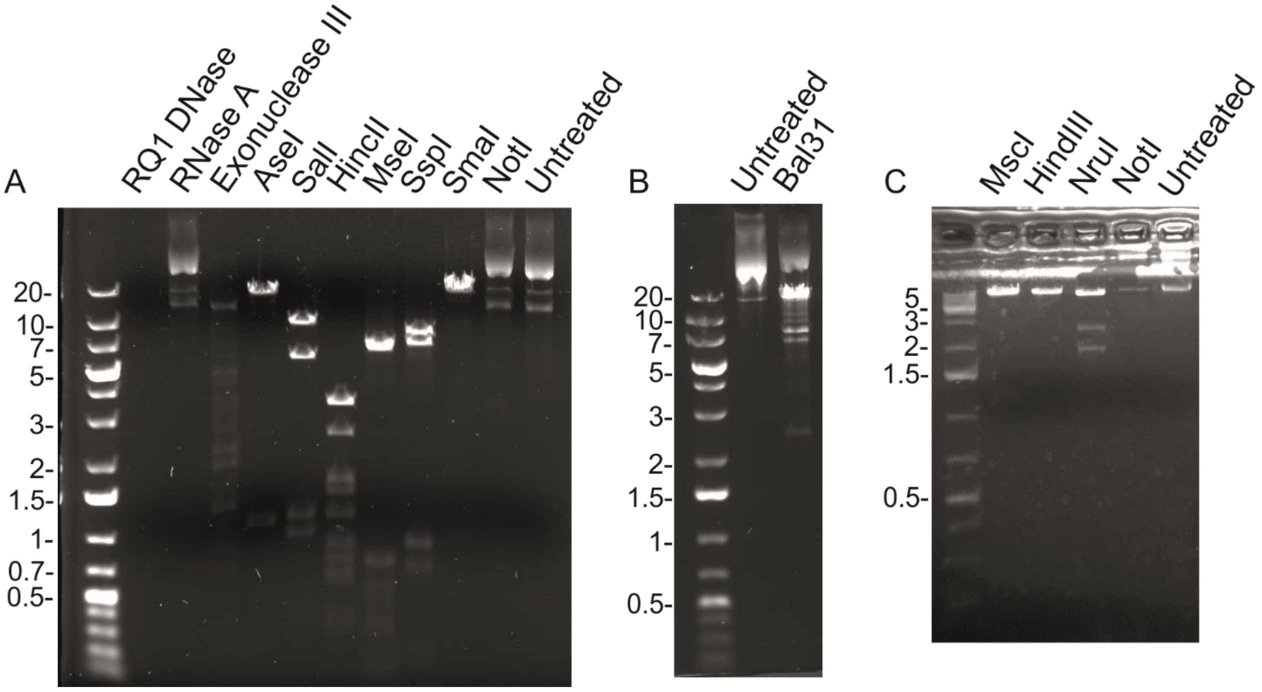

Figure S2. HHPV4 genome treated with nucleases or restriction enzymes, analysed in agarose gels stained with ethidium bromide. Molecular mass standards (kb) are shown on the left of each panel.

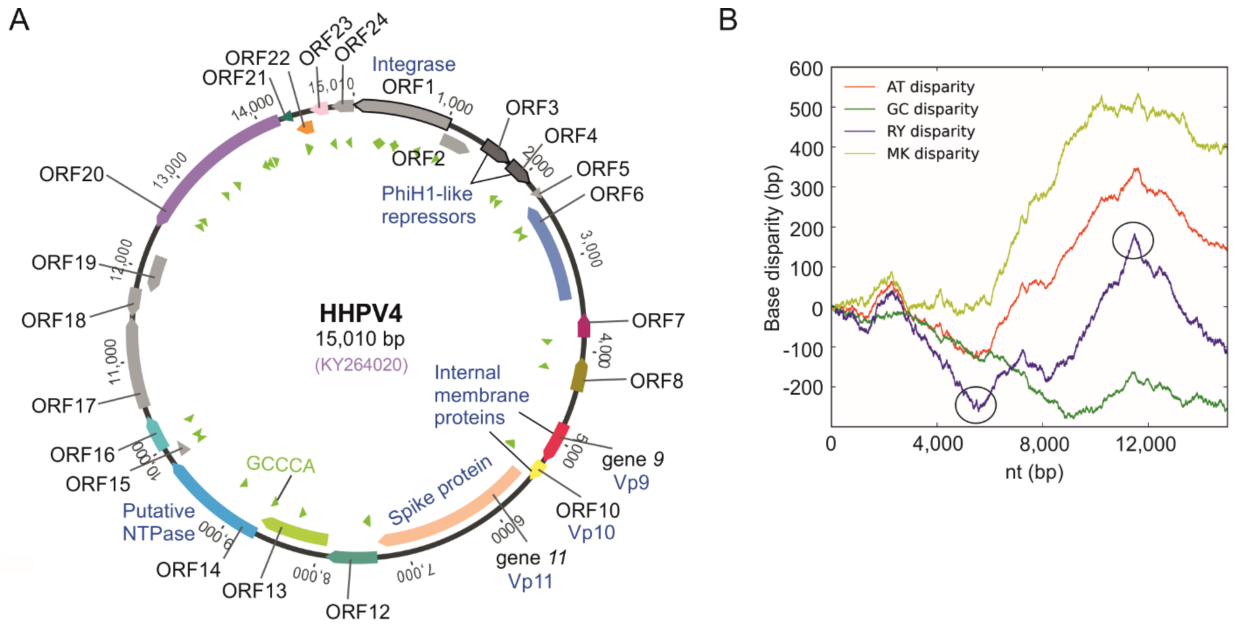

Figure S3. (**A**) Identified genes and predicted ORFs in the HHPV4 genome. ORFs are in the same colours as in Figure 5. GCCCA motifs identified on both strands are shown as green arrow heads. (**B**) HHPV4 genome sequences are represented as RY (the distribution of purine versus pyrimidine nucleotides), AT (adenine over thymine), GC (guanine over cytosine), and MK (amino bases (A and C) over keto bases (G and T)) disparity curves obtained using the Z-curve method (Ori-Finder 2 program (Luo et al., 2014)). Circles indicate the transition sites in the RY curve, which may correspond to the genome replication origin and terminus.

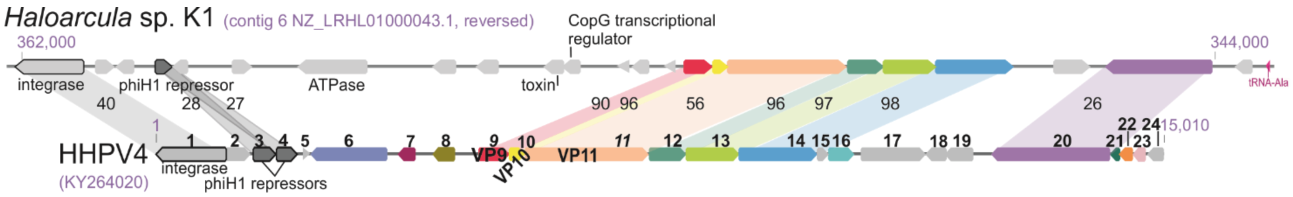

Figure S4. A putative HHPV4-like provirus in the genome of *Haloarcula* sp. K1. ORFs/genes are represented as arrows. ORFs/genes are numbered (gene numbers are in italics) and virion proteins (VPs) are marked for HHPV4. Similar ORFs/genes are in the same colour, and amino acid identities (%) of (putative) proteins are shown in between the sequences.

Table S1. HHPV4 virus purification statistics [a].

	Titer (pfu/mL)	In total (pfus)	Recovery (%)
Agar stock	1.7×10^{11}	1.0×10^{14}	100
PEG precipitated viruses	9.1×10^{12}	9.6×10^{13}	96
1× light scattering zone (10-40 % sucrose gradient)	1.1×10^{12}	5.4×10^{13}	54
2× light scattering zone (20-60 % sucrose gradient)	5.6×10^{11}	2.3×10^{13}	23
Concentrated 2× purified virus	8.1×10^{13}	1.5×10^{13}	15

a. purification of ~600 ml agar stock using HHPV4 buffer and a Sorvall AH629 rotor.

Table S2. Origin recognition box (ORB) sequences found with the Ori-Finder 2 program searching for patterns that are specific for *Halobacteriaceae*.

Start-stop, nt	*p*-value[a]	Matched sequence
2147–2128	4.3×10^{-4}	CCTCCTTATTGTAAGAAGAA
3533–3514	6.7×10^{-5}	CCCCTGCGTTTCTGGATGAA
4059–4040	2.1×10^{-4}	CCACGGGGTTGCAGCTCGAA
4086–4067	3.7×10^{-5}	GCTCTTGGTTTCATCTGAGG

a. Statistical threshold (*p*-value) used for motifs search is 1E–03. The *p*- values are generated by FIMO pipeline, which is a tool in MEME suite (http://meme-suite.org/doc/fimo.html).

Table S3. HHPV4 predicted ORFs and identified genes.

ORF/ gene	Direction[a]	Start-Stop, nt	GC[b], %	ORF product	No of aa res./ MW, kDa[c]	Calc. pI[d]	TMH/Cons. Domains/ signal pept./ coiled-coil(s)[e]	Predicted function	Corresponding viral (putative) proteins	Aa identity[f], %	Aa similarity[g], %
ORF1	R	1038–1	58.7	putative protein 1	345/39.6	5.64	-/yes/-/-	Integrase	SNJ2 ORF1 product (integrase)	70.8	85.4
ORF2	F	1021–1386	56.6	putative protein 2	121/13.5	9.98	-/-/-/-				
ORF3	F	1453–1779	52	putative protein 3	108/12.1	4.56	-/yes/-/-	PhiH1-like repressor	SNJ2 ORF4 product (phiH1-like repressor)	29.1	45.5
ORF4	F	1797–2090	55.1	putative protein 4	97/11.3	9.61	-/yes/-/-	PhiH1-like repressor	SNJ2 ORF4 product (phiH1-like repressor)	30.6	41.8
									BJ1 gp20	28	43
ORF5	F	2184–2300	41	putative protein 5	38/4.4	4.14	-/-/-/-				
ORF6	R	3431–2286	45.6	putative protein 6	381/44.2	4.69	-/yes/-/yes	Restriction endonuclease	HHPV3 putative protein 15	56.1	57.6
ORF7[h]	R	3853–3599	51.4	putative protein 7	84/10.0	5.17	-/-/-/-		HHPV3 putative protein 16	100	100
ORF8	R	4418 –4089	38.8	putative protein 8	109/12.6	4.06	-/-/-/-		HHPV3 putative protein 17	100	100
gene *9*	F	4802–5260	60.6	VP9	152/15.6	4.06	4/ -/-/-	Internal membrane protein	HHPV3 VP1	100	100
									HRPV-3 VP1	27.1	41.6
									HGPV-1 VP2	25	40.4
									HRPV-1 VP3	22.6	37.7
									HHPV-1 VP3	18.8	31.8
									HRPV-6 VP4	22.6	37.2
									HRPV-2 VP4	20.9	35
									HHPV-2 gp3	18.1	30.2
									SNJ2 VP12	20.2	36.9
ORF10	F	5266–5496	66.2	VP10	76/7.7	4.76	1/-/-/-		HHPV3 putative protein 2	100	100
gene *11*	F	5493–7334	57.2	VP11	613/65.3	4.52	2/yes/yes/yes	Spike protein	HHPV3 VP3	100	100
									HHPV-1 VP4	19.5	31
									HRPV-6 VP5	20.1	32.2
									HRPV-2 VP5	19.3	29.6
									HRPV-1 VP4	23.7	37.9
									HRPV-3 VP2	19.7	31.4

									HGPV-1 VP4	22	35
									His2 VP1 (gp29)	32.6	48.3
									HHPV-2 gp4	22.4	37
									SNJ2 VP13	21.7	34.2
ORF12	F	7342–7893	63.4	putative protein 12	183/19.2	4.46	1/yes/yes/-		HHPV3 putative protein 4	100	100
									HHPV-1 ORF5 product	30.8	47.2
									HRPV-6 ORF6 product	24.4	37.3
									HRPV-2 ORF6 product	24.9	38.3
									HRPV-1 ORF6 product	26.7	45.6
									HRPV-3 ORF3 product	25.8	41.9
									HGPV-1 ORF5 product	21.4	31.6
									His2 ORF30 product	19.1	32.2
									HHPV-2 gp5	23.8	37.7
									SNJ2 ORF14 product	24.1	40.2
ORF13	F	7890–8684	62.3	putative protein 13	264/29.8	4.63	2/yes/-/-		HHPV3 putative protein 5	100	100
									SNJ2 ORF15 product	36.2	53.2
									HHPV-1 ORF6 product	20	33.8
									HRPV-6 ORF7 product	22.7	38.6
									HRPV-2 ORF7 product	23.9	39.8
									HRPV-1 ORF7 product	24.4	37.8
									HRPV-3 ORF4 product	36.3	51.4
									HGPV-1 ORF6 product	18.4	30.4
									His2 ORF31 product	19.9	34.6
									HHPV2 gp6	19.3	34.5
ORF14	F	8677–9849	58.9	putative protein 14	390/44.2	4.83	-/yes/-/-	NTPase	HHPV3 putative protein 6	100	100
									SNJ2 ORF17 product	40.3	56.8
									HHPV-1 ORF7 product	20.5	33
									HRPV-6 ORF8 product	23.9	37.2
									HRPV-2 ORF8 product	22.4	35.2
									HRPV-1 ORF8 product	19	31.9
									HRPV-3 ORF5 product	47.9	65.2
									HGPV-1 ORF7 product	22.8	38.4
									His2 ORF33 product	30.7	47.3
									HHPV2 gp7	20.4	33.8
ORF15	F	9846–10,013	61.9	putative protein 15	55/6.1	6.03	-/-/-/-				
ORF16	F	10,010–10,399	53.1	putative protein 16	129/15.2	9.58	-/-/-/-		HHPV3 pp7	78.3	90.7
									SNJ2 ORF18 product	51.5	70

									HRPV-3 ORF6 product	44.9	59.6
									HGPV-1 ORF9 product	33.6	51.1
									HHIV-2 putative protein 38	22.4	36.5
ORF17	F	10,500-11,465	38.6	putative protein 17	321/36.3	4.62	2/-/-/-				
ORF18	R	11,768-11,466	40.9	putative protein 18	100/11.8	4.4	-/-/-/-				
ORF19	R	12,171-11,752	44.8	putative protein 19	139/15.6	6.27	-/-/-/-				
ORF20	R	14,209-12,446	61.6	putative protein 20	587/67.6	5.04	-/-/-/yes		HHPV3 putative protein 11	100	100
									SNJ2 ORF19 product	39.8	53.9
									HRPV-3 ORF9 product	25.4	41.5
									HGPV-1 ORF14 product	27.3	41.8
ORF21	R	14,355-14,212	59	putative protein 21	47/5.5	4.25	-/-/-/yes		HHPV3 putative protein 12	100	100
ORF22	R	14,549-14,352	63.1	putative protein 22	65/7.2	6.18	-/yes/-/-	RNA polymerase sigma factor, sigma-70 family	HHPV3 putative protein 13	100	100
ORF23	R	14,734-14,546	61.4	putative protein 23	62/7.1	4.67	-/-/-/-		HHPV3 putative protein 14	100	100
ORF24	R	15,008-14,769	55.8	putative protein 24	79/8.8	11.5	-/-/-/-				

a. F, forward; R, reverse.
b. GC content.
c. Number of amino acid residues and calculated molecular weight.
d. Calculated isoelectric point.
e. Predicted transmembrane helix(-ces)/conserved domain(s)/signal peptide(s)/coiled-coil region(s).
f. Amino acid identity.
g. Amino acid similarity.
h. HHPV4 ORFs/genes that are 100% identical to the corresponding ORFs/genes of HHPV3 are highlighted with red.

Table S4. Putative conserved domains detected in the HHPV4 (putative) proteins.

**Protein**	**Size, aa**[a]	**Interval, aa**[b]	**Description**[c]	**Accession**[d]	**E-value**
Putative protein 1	345	135–316	DNA breaking-rejoining enzymes, C-terminal catalytic domain	cd00397	2.22E–07
		24–97	Phage integrase, N-terminal SAM-like domain	pfam02899	9.17E–07
		127–319	Phage integrase family;	pfam00589	5.99E–06
		17–241	Tyrosine recombinase XerC	TIGR02224	1.69E–08
		17–178	Site-specific tyrosine recombinase XerC	PRK00236	1.92E–06
		25–177	Site-specific recombinase XerD	COG4974	9.77E–06
Putative protein 3	108	15–56	MarR family (MarR proteins are involved in a non-specific resistance system)	pfam12802	2.81E–05
		14–57	Arsenical Resistance Operon Repressor and similar prokaryotic, metal regulated homodimeric repressors (helix-turn-helix bacterial transcription regulatory proteins)	cd00090	2.99E–04
		14–94	Helix-turn-helix multiple antibiotic resistance protein	smart00347	8.60E–08
		14–85	DNA-binding transcriptional regulator, MarR family	COG1846	5.95E–05
Putative protein 4	97	12–57	Winged helix-turn-helix DNA-binding proteins	pfam13412	3.52E–03
		12–72	Helix-turn-helix ASNC type	smart00344	6.30E–03
		12–51	DNA-binding transcriptional regulator, Lrp family	COG1522	0.01
Putative protein 6	381	172–221	Dam-replacing family	pfam06044	8.43E-18
		276–329	HNH endonuclease	pfam13391	2.08E-13
		272–319	HNH nucleases	smart00507	7.26E–05
		273–319	HNH nucleases	cd00085	8.35E–05
		198–262	Domain of unknown function (DUF4404)	pfam14357	0.05
VP11	613	202–267	Syntaxin N-terminal domain	smart00503	2.70E–03
Putative protein 12	183	5–64	Domain of unknown function (DUF4366)	pfam14283	7.66E–03
Putative protein 13	264	32–76	Putative ammonia monooxygenase	pfam05145	0.01
Putative protein 14	390	134–262	AAA-ATPases associated with a variety of cellular activities	smart00382	0.03
Putative protein 17	321	95–178	Protein of unknown function (DUF2393)	pfam09624	0.01
		71–112	Prolipoprotein diacylglyceryl transferase	PRK12437	0.04
Putative protein 20	587	439–562	Exocyst complex component Sec10	pfam07393	0.03
		425–566	Chromosome segregation protein	PRK02224	0.03
Putative protein 22	65	13–64	DNA-binding transcriptional regulator, CsgD family	COG2771	6.66E–06
		14–64	Helix-turn-helix, Lux Regulon	smart00421	1.21E–05
		17–60	Sigma-70, region 4	pfam08281	3.99E–05
		17–64	C-terminal DNA-binding domain of LuxR-like proteins	cd06170	1.81E–03
		14–60	RNA polymerase sigma factor, sigma-70 family	TIGR02937	2.38E–05
		14–59	DNA-directed RNA polymerase specialized sigma subunit, sigma24 family	COG1595	2.99E–05

a. Total number of amino acid residues.

b. Interval, amino acid residues.

c. All hits were found using BLASTp (Delta-BLAST), search dated 22.11.2016. E-value threshold is 0.05.
d. Accession number in the NCBI’s conserved domain database.
